# High Resolution Melt Assays to Detect and Identify *Vibrio parahaemolyticus*, *Bacillus cereus*, *Escherichia coli*, and *Clostridioides difficile* Bacteria

**DOI:** 10.3390/microorganisms8040561

**Published:** 2020-04-14

**Authors:** Allison C. Bender, Jessica A. Faulkner, Katherine Tulimieri, Thomas H. Boise, Kelly M. Elkins

**Affiliations:** Chemistry Department, Towson University, 8000 York Road, Towson, MD 21252, USA

**Keywords:** dysbiosis, pathogen, *Escherichia coli*, *Vibrio parahaemolyticus*, *Clostridioides difficile*, *Bacillus cereus*, polymerase chain reaction, melt curves, triplex assay, screening

## Abstract

Over one hundred bacterial species have been determined to comprise the human microbiota in a healthy individual. Bacteria including *Escherichia coli*, *Bacillus cereus*, *Clostridioides difficile*, and *Vibrio parahaemolyticus* are found inside of the human body and *B. cereus* and *E. coli* are also found on the skin. These bacteria can act as human pathogens upon ingestion of contaminated food or water, if they enter an open wound, or antibiotics, and environment or stress can alter the microbiome. In this study, we present new polymerase chain reaction (PCR) high-resolution melt (HRM) assays to detect and identify the above microorganisms. Amplified DNA from *C. difficile*, *E. coli, B. cereus*, and *V. parahaemolyticus* melted at 80.37 ± 0.45 °C, 82.15 ± 0.37 °C, 84.43 ± 0.50 °C, and 86.74 ± 0.65 °C, respectively. A triplex PCR assay was developed to simultaneously detect and identify *E. coli*, *B. cereus*, and *V. parahaemolyticus*, and cultured microorganisms were successfully amplified, detected, and identified. The assays demonstrated sensitivity, specificity, reproducibility, and robustness in testing.

## 1. Introduction

Healthy individuals have a diverse microbiota and contain over one hundred bacterial species [1]. Bacteria are found all over and inside of the human body [1,2]. *Escherichia coli*, *Bacillus cereus*, *Clostridioides difficile*, and *Vibrio parahaemolyticus* are found inside of the human body and *B. cereus* and *E. coli* are also found on the skin. *E. coli*, *B. cereus*, and *V. parahaemolyticus* can act as human pathogens upon ingestion of contaminated food or water, if they enter an open wound, or antibiotics, and environment or stress can alter the microbiome [3]. *C. difficile* has been most problematic in individuals with dysbiosis or in hospital settings, but recent research indicates that contaminated food products with spores may also lead to *C. difficile* infection [4,5]. These and other bacteria are listed by the United States Center for Disease Control and Prevention (CDC) as notable foodborne pathogens that cause the majority of such illness, along with other pathogens [2]. 

The CDC estimates that 30% of the population in industrialized countries suffers from illness caused by foodborne pathogens in contaminated food and drinking water [2]. In 2005, the CDC reported that there were 76 million cases of foodborne diseases annually, resulting in 325,000 hospitalizations and 5000 deaths annually in the USA [2]. The World Health Organization (WHO) reports 17 billion cases of diarrheal disease annually and an estimated 2.2 million deaths [6]. In the United States, there are an estimated 211–375 million cases each year leading to 1.8 million hospitalizations and 3100 deaths [7].

The cause of *E. coli* and *V. parahaemolyticus* infections is often difficult to determine but the high morbidity and mortality rates lead to a major health burden for services and infrastructure. Public health laboratories need to identify and trace outbreaks caused by microorganisms [8]. It is common for labs to perform stool cultures to diagnose and treat bacterial gastroenteritis [8]. While other pathogens cause as many illnesses, *E. coli* and *V. parahaemolyticus* are among those that are more likely to cause illness that leads to hospitalization [1,2]. *V. parahaemolyticus* causes acute gastroenteritis and can cause septicemia [9]. These species can be spread by contamination of food as a result of improper food preparation and negligence to safety precautions such as hand washing [2]. Illness can also be caused by eating raw food or undercooked food [9]. Cases are underreported and routine identification of the species causing foodborne illness is not clinically performed. 

The bacteria can also cause skin infections as a result of exposure to tainted water or soils [2]. *V. parahaemolyticus* can cause skin infections when a cut or opening in the skin is exposed to contaminated water through swimming, boating, and other outdoor activities [2]. *B. cereus* is an opportunistic pathogen found on the skin and open cuts in the skin can lead to severe skin infections [2]. Upon taking antibiotics, *C. difficile* can overpopulate and cause dysbiosis of the normal gut flora leading to severe diarrhea and pseudomembranous colitis and in severe cases, it can lead to a life-threatening infection leading to a colon perforation and septic shock, among others [1,2].

Bacterial detection and identification is a well-researched area in the literature due to the importance of the applications in medicine and public health. Over the years, several methods have been developed to detect and identify bacterial species found to contaminate food products and cause infections [10,11]. Several papers and reports have been written on the determination of bacterial strains and serogroups [12,13,14,15]. The methods include bacterial culture followed by staining techniques, evaluation of morphology using microscopy, and enzymatic activity assays, immunological assays, and polymerase chain reaction (PCR) based methods [2,16,17,18]. A drawback to bacterial culture is that it is slow and not all species can be cultured. Immunological assays include the latex agglutination test, enzyme-linked immunosorbent assays (ELISA), lateral flow immunochromatographic tests, and immunofluorescence assays [17,18]. These highly sensitive and specific assays are used to detect one target at a time. As a result, there has been a lot of interest in applying PCR-based methods toward the detection and identification of pathogens. PCR-based methods have been used to amplify targets for subsequent gel-based sizing assays, microarrays, SNaPshot assays, mRNA expression analysis, and genome sequencing [11,17,19,20]. 

Numerous nucleic acid-based PCR assays have been developed for microorganisms over the past several years. For example, Toma et al. [21] developed a PCR assay followed by gel electrophoresis to discriminate *E. coli* strains by band patterns. Kagkli et al. [22] published a PCR high-resolution melt (HRM) assay for *E. coli* using SYBR® Green to differentiate the top five “serogroups” using melt curves and difference plots. Harrison and Hanson [23] showed how PCR HRM can be used to differentiate between sequence types of *E. coli* with principle component analysis (PCA) of the melt curves. A real-time PCR four TaqMan probe assay was developed to detect the total and pathogenic *V. parahaemolyticus* [9]. A real-time PCR multiplex was developed to detect *C. difficile* using toxin genes tcdA and tcdB; positive samples were differentiated from negatives by analysis of cycle threshold amplification [24]. Although toxigenic and non-toxigenic *C. difficile* carry these genes, only toxigenic *C. difficile* express the potent and lethal proteins [25]. *B. cereus* and *B. thuringiensis* were differentiated by a PCR and gel assay [26]. The drawback of many PCR-based methods is that several additional preparation and procedure steps for analysis, including post-PCR gel, difference plot, or PCA analysis, are typically required. Most assays each target only one species, although some differentiate strains. Post-PCR melt assay melt temperatures can be used to detect amplicon production and identify bacterial species using real-time PCR without additional steps, analysis, or expensive dye-labeled probes [19]. 

The present work demonstrates new genetic assays to detect and identify *E. coli, B. cereus, C. difficile*, and *V. parahaemolyticus* in separate real-time PCR high resolution melt assays developed using purchased DNA standards, as well as a triplex assay to detect and identify *E. coli, B. cereus*, and *V. parahaemolyticus* simultaneously. The triplex assay was optimized using purchased DNA standards and tested with cultured pathogens.

## 2. Materials and Methods 

### 2.1. Strains, Media, and Bacterial Samples

Extracted DNA from the strains *Escherichia coli* K-12 MG1655 (700926), *Bacillus cereus* str. Frankland and Frankland (10876), *Vibrio parahaemolyticus* (17802D-5), and *Clostridioides difficile* (9689D-5) were obtained from the American Type Culture Collection (ATCC; Manassas, VA, USA). The additional bacteria and their sources used in specificity testing included *Bacillus megaterium*, *Bacillus subtilis*, *Bacillus thuringiensis*, *Citrobacter freudii*, *Enterobacter aerogenes*, *E. coli* (ATCC 25922), *Klebsiella oxytoca*, *Listeria monocytogenes*, *Micrococcus luteus*, *Salmonella enterica*, *Serratia macrescens*, *Shigella flexneri*, *Staphylococcus capitis*, and *Staphylococcus saprophyticus*, as listed in Table 1. *B. cereus and E. coli* bacterial cultures were also obtained from the Carolina Biological Supply Company (Burlington, NC, USA). *E. coli* and *B. cereus* were inoculated in Luria–Bertani (LB) medium and grown at 37 °C. A hemocytometer and a compound light microscope were used to obtain cell counts. Human HL-60 DNA was also tested in the specificity studies and was obtained from ATCC.

### 2.2. DNA Extraction 

Genomic DNA was extracted using the QuickLyse Miniprep Kit (Qiagen, Germantown, MD, USA) according to the manufacturer’s protocol and eluted in 50 µL of water. The extracted DNA was quantified using a NanoDrop 2000 instrument (ThermoFisher, Frederick, MD, USA) using the single stranded nucleic acid option.

### 2.3. Primer Design

PCR primers were designed to produce amplicons of varied lengths for each species, as described previously [19]. Briefly, target regions were selected in consultation with the literature that indicated unique gene coding sequences in each species (Table 2). Primers were designed to amplify a portion of the region and were adjusted to yield amplicons that would melt at temperatures that would be unique for each species [19]. The primers were purchased from Integrated DNA Technologies (IDT DNA; Coralville, IA, USA).

### 2.4. Multiplex Assay Development 

The lyophilized primers were reconstituted with nuclease-free water, quantified using the NanoDrop 2000 using the oligo option, and diluted to 5 µM working stocks. The primers were added to 2× LightScanner master mix (BioFire Defense, Murray, UT, USA) to prepare a PCR reaction mix for testing. Each primer set was tested singly with the 5 µM stock primers in a 1:1 ratio using 1 µL of each primer from the stock, 1 µL of 1 ng/µL of DNA, 8 µL of master mix, and 9 µL of nuclease-free water in a 20 µL reaction to determine amplification of the target. The amplified DNA was sized using a 3% agarose gel to determine that the correct sized target was produced. For the multiplex assay, primer ratios were adjusted so that each species would be amplified with a similar level of product. The final concentration conditions for the multiplex were 1 µL each of the 5 µM forward and reverse primer stocks for *E. coli* and *V. parahaemolyticus*, and 0.5 µL each of the 5 µM forward and reverse primer stocks for *B. cereus*. 

### 2.5. PCR Reaction Conditions

The input DNA concentration was 1 ng/µL except where noted in specificity studies, in which a serial dilution of standard DNA was prepared from 10 to 0.01 ng/µL and the indicated quantity was inputted. The DNA was amplified using a Rotor-Gene Q real-time PCR instrument (Qiagen, Germantown, MD, USA) using the following settings: 95 °C for 2 min initial denaturation, 29 cycles of 95 °C for 10 s denaturation, 66 °C for 10 s primer annealing, and 72 °C for 10 s primer extension detecting HRM. For the *C. difficile* assay, the following temperature program was used: 95 °C for 2 min initial denaturation, 40 cycles of 95 °C for 20 s denaturation, 62 °C for 20 s primer annealing with touchdown of 2 cycles at 0.5°, and 72 °C for 20 s primer extension detecting HRM. Melt analysis was performed using the following settings directly after amplification: 65 to 95 °C melt increasing by 0.2 °C in 2 s intervals detecting HRM.

### 2.6. Sensitivity and Specificity Testing

The sensitivity of the assays was tested by serial dilution of the standard DNA to yield DNA concentrations of 10, 5, 1, 0.5, 0.1, 0.05, and 0.01 ng/µL. One nanogram of *E. coli* DNA is 196,679 cells based upon the size of the *E. coli* genome 4,639,221 bp [27]. The specificity of each primer set was tested using 1 ng input of the eighteen species of bacteria and human DNA described above.

Sensitivity of the *E. coli* qPCR assay was tested using LB and commercial apple cider inoculated with 10^8^ to 10^0^ *E. coli* cells per milliliter or colony forming units (CFU) prepared by serial dilution. The samples were extracted using the QuickLyse Miniprep Kit and eluted in 20 µL and 2 µL of the extract was amplified using the qPCR assay run for 40 cycles. The limit of quantitation (LOQ) was determined using the amplification curves, and the limit of detection (LOD) was determined using the melt curves.

### 2.7. Evaluation of Multiplex Assay for Bacterial Identification 

The multiplex PCR assay was used to analyze simulated case scenario samples from DNA extracted from purchased bacterial cultures as described above. 

## 3. Results

**Figure 1:**
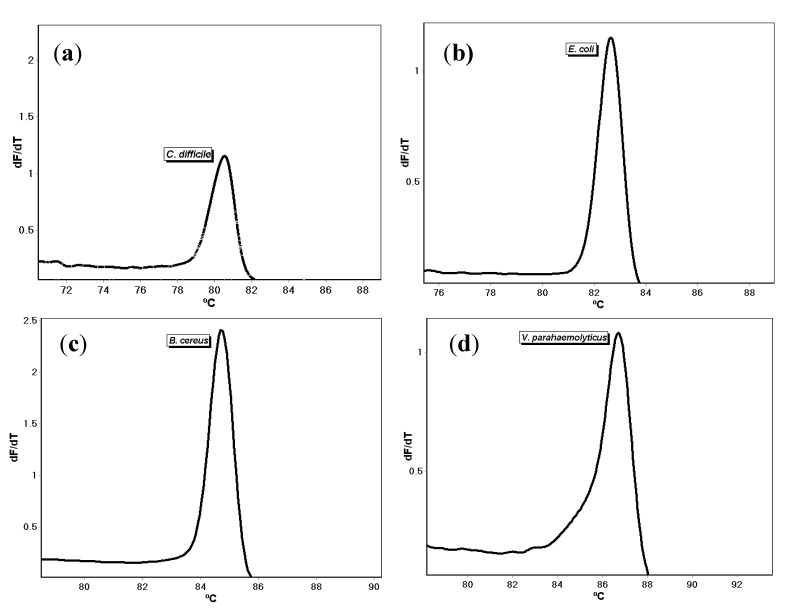
Single melt curves using individual primer set for (**a**) *C. difficile*, (**b**) *E. coli*, (**c**) *B. cereus*, and (**d**) *V. parahaemolyticus.*

Single melt PCR assays for *E. coli, B. cereus, C. difficile*, and *V. parahaemolyticus* were developed with primers targeting the *yedN*, *cmk*, *tcdA*, and *tlh* genes, respectively (Table 2). The primers were prepared, and the PCR reactions were performed as described. Amplification of one nanogram of standard DNA purchased from ATCC resulted in different melt temperatures of 80.37 ± 0.45 °C (*n* = 4), 82.15 ± 0.37 °C (*n* = 4), 84.43 ± 0.50 °C (*n* = 4), and 86.74 ± 0.65 °C (*n* = 4) for *C. difficile*, *E. coli, B. cereus*, and *V. parahaemolyticus*, respectively (Figure 1). To determine that the intended DNA targets were amplified, post-PCR gel electrophoresis was performed and revealed the expected fragment sizes. The no-template control samples did not amplify. The amplicons produced in all of the assays were re-melted to determine the robustness of the melt detection with very low standard deviations of ±0.06 °C, ±0.02 °C, and ±0.02 °C, for *E. coli*, *B. cereus*, and *V. parahaemolyticus*, respectively.

**Figure 2:**
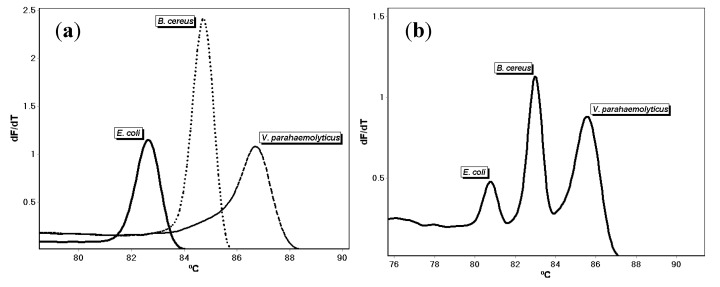
(**a**) Polymerase chain reaction (PCR) melt curves for *E. coli*, *B. cereus*, *and V. parahaemolyticus* DNA tested individually using the triplex assay primer mix. (**b**) Melt curves of *E. coli, B. cereus*, and *V. parahaemolyticus* mixture detected together using the triplex assay.

The primers for *E. coli, B. cereus*, and *V. parahaemolyticus* were combined and optimized to develop a triplex assay as described in the Materials and Methods. Testing using one nanogram each of ATCC DNA produced three amplicons that melt at 81.48 ± 0.71 °C (*E. coli*; *n* = 7), 83.69 ± 0.70 °C (*B. cereus*; *n* = 7), and 86.13 ± 0.57 °C (*V. parahaemolyticus*; *n* = 7), respectively (Figure 2). A no-template control did not amplify. These values reflect the assays being repeated several times on different days by the same researcher and reproduced by other researchers in the lab.

The triplex assay was used to test DNA extracted from *E. coli* and *B. cereus* bacteria cultured in LB media. *V. parahaemolyticus* was not tested as we were not able to test it in our lab. The extracted DNA for *E. coli* and *B. cereus* amplified and melted at 82.74 ± 0.44 °C (*n* = 6) and 84.77 ± 0.36 °C (*n* = 4), respectively.

**Figure 3:**
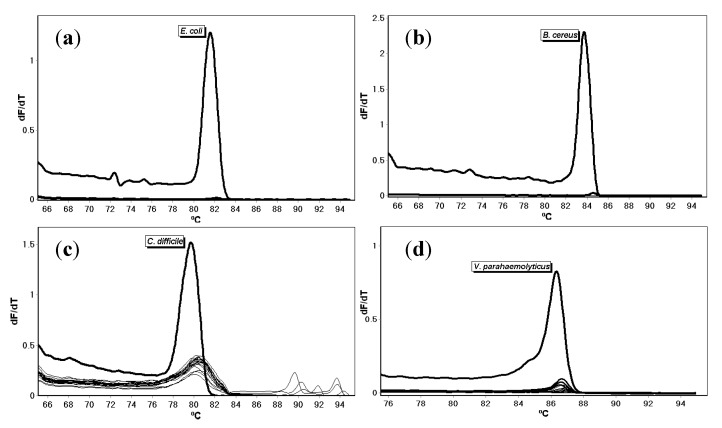
Melt curves for (**a**) *E. coli*, (**b**) *B. cereus*, (**c**) *C. difficile*, and (**d**) *V. parahaemolyticus* primers tested with DNA from 18 bacterial species and human DNA.

Specificity testing of each primer set conducted with DNA extracted from eighteen species of non-target bacteria in separate reactions (Table 1) did not lead to the production of an amplicon as detected with melt temperature analysis (Figure 3). Only the intended targets amplified. The no-template control and human DNA did not amplify.

**Figure 4:**
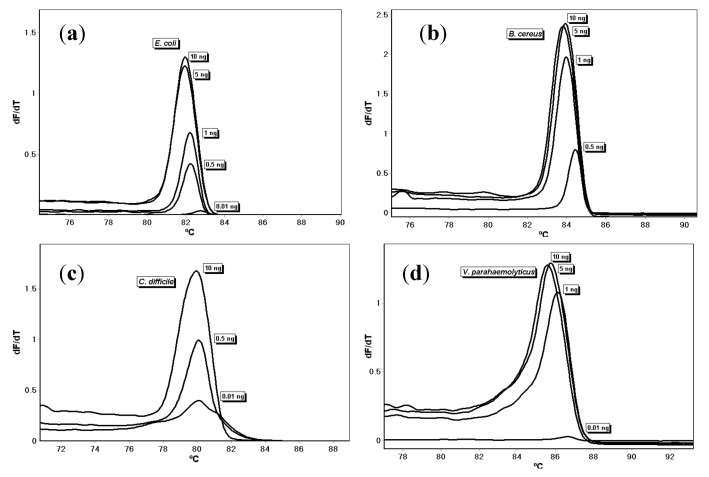
Melt curves of DNA dilution series for (**a**) *E. coli*, (**b**) *B. cereus*, (**c**) *C. difficile*, and (**d**) *V. parahaemolyticus*.

A DNA dilution series to determine sensitivity demonstrated that the assays were sensitive to 0.5 ng of input DNA for *E. coli*, *B. cereus*, and *C. difficile*, and 1.0 ng of input DNA for *V. parahaemolyticus* (Figure 4). Overall, the melt temperatures were 82.41 ± 0.43 °C, 84.04 ± 0.28 °C, 80.23 ± 0.24 °C, and 85.81 ± 0.26 °C, for *E. coli*, *B. cereus, C. difficile*, and *V. parahaemolyticus*, respectively.

Serial dilutions were created from *E. coli* cultures ranging from 100 million cells/mL to 1 cell/mL (CFU). DNA was extracted from each dilution using the QuickLyse Miniprep Kit and quantitated with the *E. coli* qPCR assay run for 40 cycles. The PCR HRM limit of detection for identification for *E. coli* cultured in LB was 1000 cells/mL based on the melt peak and 1 cell/mL limit of quantitation based on the amplification cycle threshold. The PCR melt limit of detection for identification for *E. coli* in the cider was 1 million cells/mL. 

## 4. Discussion

We developed the PCR primers used in the assays based upon genes with sequences unique to the species reported in the literature. As in Ward and Bej [9], our PCR primers for *V. parahaemolyticus* target the *tlh* gene encoding the thermostable direct hemolysin virulence factor [28]. Our *C. difficile* primers target the *tcdA* gene encoding a toxin [24,29]; the gene was also probed with a fluorescence-based multiplex real-time PCR assay [24]. The primers for *B. cereus* target the cytidylate kinase (*cmk*) gene described in a paper showing evidence that *B. anthracis*, *B. cereus*, and *B. thuringiensis* are genetically one species [30]. The *E. coli* primers target the *yedN* pseudogene region [19], for which no information was available when they were designed. More recently, the region has been ascribed to a “putative type III secreted effector” [31]. The putative primers were submitted to the basic local alignment search tool (BLAST) and the option to search “somewhat similar sequences (blastn)” was selected with the default settings [32]; we moved forward with primer sequences that demonstrated high specificity and selectivity. The primer sequences were synthesized and purchased from a reputable supplier, IDT DNA (Coralville, IA, USA). 

Developmental validation testing of the new PCR melt assays demonstrates that *E. coli*, *B. cereus*, *C. difficile*, and *V. parahaemolyticus* have unique melt temperatures that were repeatable and reproducible by multiple investigators. This, along with the confirmation of amplicon DNA fragment size via gel electrophoresis, show that the primers are accurate in their amplification, and produce different melt temperatures for each species. To ensure that the primers are specific to only the intended DNA species, specificity testing using DNA from 18 bacterial species revealed that the primers amplified and produced the unique melt curve with only the targeted species and not the other DNA, the human DNA, or no-template control. Although the second hit in BLAST for the forward primer for *B. cereus* was *B. thuringiensis*, the primer set did not amplify *B. thuringiensis* under the PCR conditions we used in our assays. Detection of the *tcdA* gene in *C. difficile* indicates that the primers were specific for the toxigenic bacteria; non-toxigenic bacteria that carry the genes, even if they do not express the proteins, would also be detected [25]. To determine the sensitivity of the primers, a dilution series was performed using varying concentrations of input DNA. Detectable amplification using the melt curves was achieved using 0.5 ng for *E. coli*, *B. cereus*, and *C. difficile*, and 1 ng for *V. parahaemolyticus* when using the 29-cycle amplification PCR temperature program. The melt temperatures shifted to a higher melt temperature as the concentration of input DNA decreased; the assays are sensitive to input DNA concentration. Extending the cycling to 40 cycles increased the sensitivity but led to non-specific amplification. To test the robustness of the melt curve feature, the PCR amplicons were re-melted; we observed a very low standard deviation on the temperatures upon re-melting compared to the originals. These results of repeatability, reproducibility, specificity, sensitivity, and re-melt testing validate the primers, providing robust assays for the detection and identification of *E. coli*, *B. cereus*, *C. difficile*, and *V. parahaemolyticus*.

Multiplex testing using the *E. coli*, *B. cereus*, and *V. parahaemolyticus* primer triplex demonstrates that one assay that can simultaneously detect and identify all three species and was successful in amplifying the two bacteria tested and producing melt temperatures at the expected region. This triplex assay is advantageous over other methods of bacterial detection because it is fast, affordable, and reliable and can simultaneously detect and identify three bacterial species implicated in human dysbiosis and foodborne illness in one assay from DNA extracted from a sample. The assay is easy to interpret and does not require highly trained microbiologists to identify the pathogens by color, shape, enzyme reactivity, staining patterns, and morphology, nor does it require sophisticated software for PCA. While the assay does not differentiate serogroups, it does have the ability to detect and identify three different bacteria that are known to cause bacterial gastroenteritis and dysbiosis. The *C. difficile* toxins TcdA and TcdB have also been shown to cause damage to colon tissue [29]. The PCR melt assay approach has been applied previously to food-borne pathogens; our group developed a triplex assay for *E. coli*, *S. enterica*, and *S. flexneri* in 2016 [19]. Nucleic acid-based assays are valuable for detecting microorganisms in food, water, and stool samples so that cases can be appropriately logged and treated [6,7,8,9]. The PCR melt assays are more selective than many dipstick tests and determine which, if any, of the bacteria are present in the sample at a similar reagent cost. Dipstick tests generally have specificity issues, although a 24 h version of the test has recently been adapted to detect *V. cholerae* [33]. A drawback to the adaptation though is that it greatly increases the time needed to process samples. The triplex assay can be run in approximately 75 minutes for PCR and melt. 

The *E. coli* assay was used to quantitate *E. coli* inoculated in LB and apple cider. The results demonstrate qPCR assay is sensitive and the assay can detect and quantify *E. coli* in the food matrix, but the detection limit was higher in apple cider than in LB. The triplex assay tested with *E. coli* and *B. cereus* cultured in LB demonstrates that both bacteria were detectable and identifiable. 

## 5. Conclusions

While humans coexist with bacteria in our environment, on the skin and in the body, an imbalance in the bacteria can cause significant illness. We have developed new PCR melt assays to detect and identify *C. difficile*, *E. coli*, *B. cereus*, and *V. parahaemolyticus*, and have presented the results of our developmental validation. Our triplex PCR assay simultaneously detects and identifies *E. coli*, *B. cereus*, and *V. parahaemolyticus*. Future studies using clinical samples in collaboration with medical researchers are needed to develop thresholds for detecting normal levels of the bacteria and levels indicating disease. The assay could be employed to trace variation over time or upon environmental changes. Sampling of additional food products could be performed to detect and identify tainted items. The recently published integrative human genome project data and resources is a rich resource for future microorganism research and disease studies [34,35,36]. 

## Figures and Tables

**Table 1:** Bacterial species and source used in assay development and specificity studies.

Bacterium	Source
*Bacillus cereus* str. Frankland and Frankland	ATCC (10876)
*Bacillus megaterium*	TU Biology Department
*Bacillus subtilis* str. Ehrenberg Cohn Str. 168	ATCC (23857)
*Bacillus thurigiensis* serovar Israelensis	ATCC (35646)
*Citrobacter freudii*	Carolina Biological
*Clostridioides difficile* 90556-M6S	ATCC (9689D-5)
*Enterobacter aerogenes*	TU Biology Department
*Escherichia coli* K-12 MG1655	ATCC (700926)
*Escherichia coli* (Migula) Castellani and Chalmers	ATCC (25922)
*Klebsiella oxytoca* MsA1	ATCC
*Listeria monocytogenes* str. EGD	ATCC (BAA-679D-5)
*Micrococcus luteus*	Midwest Culture Service
*Salmonella enterica* serovar Typhimurium str. LT2	ATCC (700720)
*Serratia macrescens*	Carolina Biological
*Shigella flexneri* str. 2547T	ATCC (29903)
*Staphylococcus capitis* subsp. capitis Kloos and Schleifer	ATCC (35661)
*Staphylococcus saprophyticus*	Wards Natural Science
*Vibrio parahaemolyticus* str. EB 101	ATCC (17802D-5)

**Table 2:** Primer sequences used to amplify each species.

Target Specie	Primer Sequence (5’–3’)	Gene Target	Amplicon Length (bp)	Reference
*C. difficile*	F-GTTAGCATCCGTATTAGCAGGTGC R-ACAGCTATGGGTGCGAATGG	tcdA	135	This study
*E. coli*	F-TCCTGGATTGAGGTGCTTTATC R-CTACGGAGACCTGGGTAATTCC	yedN	142	Elkins et al. [19]
*V. parahaemolyticus*	F-ACTGGATTTCGCTTTGCCCTCAATGA R-GTTCTGAGTTCGATAACCTCTTGTGTGGATTAAG	tlh	146	This study
*B. cereus*	F-GAAAAGTACAGTGGCAAAAGTTGTTGCG R-CGCTAACTCTTGCTGACGACGT	cmk	313	This study

## References

[1] Switai T.L., Winter K.J., Christensen S.R. (2015). Diagnosis and Management of Foodborne Illness. Am. Fam. Physician.

[2] Estimates of Foodborne Illness in the United States Centers for Disease Control and Prevention. https://www.cdc.gov/foodborneburden/estimates-overview.html.

[3] Omer M.K., Álvarez-Ordoñez A., Prieto M., Skjerve E., Asehun T., Alvseike O.A. (2018). A Systematic Review of Bacterial Foodborne Outbreaks Related to Red Meat and Meat Products. Foodborne Pathog. Dis..

[4] Candel-Pérez C., Ros-Berruezo G., Martínez-Graciá C. (2019). A review of *Clostridioides [Clostridium] difficile* occurrence through the food chain. Food Microbiol..

[5] Rohlke F., Stollman N. (2012). Fecal microbiota transplantation in relapsing *Clostridium difficile* infection. Therap Adv. Gastroenterol..

[6] Diarrhoeal Disease World Health Organization. http://who.int/mediacentre/factsheets/fs330/en/index.html.

[7] Guerrant R.L., Van Gilder T., Steiner T.S., Thielman N.M., Slutsker L., Tauxe R.V., Hennessy T., Griffin P.M., DuPont H., Sack R.B. (2001). Practice guidelines for the management of infectious diarrhea. Clin Infect. Dis..

[8] Humphries R.M., Linscott A.J. (2015). Laboratory diagnosis of bacterial gastroenteritis. Clin. Microbiol. Rev..

[9] Ward L.N., Bej A.K. (2006). Detection of *Vibrio parahaemolyticus* in Shellfish by Use of Multiplexed Real-Time PCR with TaqMan Fluorescent Probes. Appl. Environ. Microbiol..

[10] Dwivedi H.P., Jaykus L. (2011). Detection of pathogens in foods: The current state-of-the-art and future directions. Crit. Rev. Microbiol..

[11] Alahi M.E.E., Mukhopadhyay S.C. (2017). Detection Methodologies for Pathogen and Toxins: A Review. Sensors.

[12] Murasova P., Kovarova A., Kasparova J., Brozkova I., Hamiot A., Pekarkova J., Dupuy B., Drbohlavova J., Bilkova Z., Korecka L. (2020). Direct culture-free electrochemical detection of Salmonella cells in milk based on quantum dots-modified nanostructured dendrons. J. Electroanal. Chem..

[13] Sánchez-Chica J., Correa M.M., Aceves-Diez A.E., Castañeda-Sandoval L.M. (2020). A novel method for direct detection of *Bacillus cereus* toxin genes in powdered dairy products. Int. Dairy J..

[14] Jayan H., Pu H., Sun D.-W. (2020). Recent development in rapid detection techniques for microorganism activities in food matrices using bio-recognition: A review. Trends Food Sci. Technol..

[15] Sharifi S., Vahed S.Z., Ahmadian E., Dizaj S.M., Eftekhari A., Khalilov R., Ahmadi M., Hamidi-Asl E., Labib M. (2020). Detection of pathogenic bacteria via nanomaterials-modified aptasensors. Biosens. Bioelectron..

[16] Elkins K.M. (2018). Introduction to Forensic Chemistry.

[17] Priyanka B., Patil R.K., Dwarakanath S. (2016). A review on detection methods used for foodborne pathogens. Indian J. Med Res..

[18] Law J.W.-F., Mutalib N.-S.A., Chan K.-G., Lee L.-H. (2015). Rapid methods for detection of foodborne bacterial pathogens: Principles, applications, advantages and limitations. Front. Microbiol..

[19] Elkins K.M., Perez A.C.U., Sweetin K.C. (2016). Rapid and inexpensive species differentiation using a multiplex real-time polymerase chain reaction high-resolution melt assay. Anal. Biochem..

[20] Rajapaksha P., Elbourne A.J., Gangadoo S., Brown R., Cozzolino D., Chapman J. (2019). A review of methods for the detection of pathogenic microorganisms. Analyst.

[21] Toma C., Lu Y., Higa N., Nakasone N., Chinen I., Baschkier A., Rivas M., Iwanaga M. (2003). Multiplex PCR Asaay for Identification of Human Diarrhaegenic *Escherichia coli*. J. Clin. Microbiol..

[22] Kagkli D.-M., Folloni S., Barbau-Piednoir E., Van den Eede G., Van den Bulcke M. (2012). Towards a Pathogenic *Escherichia coli* Detection Platform Using Multiplex SYBR Green Real-Time PCR Methods and High Resolution Melting Analysis. PLoS ONE.

[23] Harrison L.B., Hanson N.D. (2017). High-Resolution Melting Analysis for Rapid Detection of Sequence Type 131 *Escherichia coli*. Antimicrob. Agents Chemother..

[24] Bélanger S.D., Boissinot M., Clairoux N., Picard F.J., Bergeron M.G. (2003). Rapid Detection of *Clostridium difficile* in Feces by Real-Time PCR. J. Clin. Microbiol..

[25] Natarajan M., Walk S.T., Young V.B., Aronoff D.M. (2013). A Clinical and Epidemiological Review of Non-toxigenic *Clostridium difficile*. Anaerobe.

[26] Hansen B.M., Hendriksen N.B. (2001). Detection of Enterotoxic *Bacillus cereus* and *Bacillus thuringiensis* Strains by PCR Analysis. Appl. Environ. Microbiol..

[27] Blattner F.R., Plunkett G., Bloch C.A., Perna N.T., Burland V., Riley M., Collado-Vides J., Glasner J.D., Rode C.K., Mayhew G.F. (1997). The complete genome sequence of Escherichia coli K-12. Science.

[28] Gutierrez West C.K., Klein S.L., Lovell C.R. (2013). High Frequency of Virulence Factor Genes *tdh*, *trh*, and *tlh* in *Vibrio parahaemolyticus* Strains Isolated from a Pristine Estuary. Appl. Environ. Microbiol..

[29] Chumbler N.M., Farrow M.A., Lapierre L.A., Franklin J.L., Lacy D.B. (2016). *Clostridium difficile* Toxins TcdA and TcdB Cause Colonic Tissue Damage by Distinct Mechanisms. Infect. Immun..

[30] Helgasonm E., Okstad O.A., Caugant D.A., Johansen H.A., Fouet A., Mock M., Hegna I., Kolstø A.B. (2000). *Bacillus anthracis*, *Bacillus cereus*, and *Bacillus thuringiensis*—One Species on the Basis of Genetic Evidence. Appl. Environ. Microbiol..

[31] EcoCyc. https://biocyc.org/gene?orgid=ECOLI&id=G7043.

[32] Standard Nucleotide BLAST NCBI. https://blast.ncbi.nlm.nih.gov/Blast.cgi?PAGE_TYPE=BlastSearch.

[33] Chakraborty S., Alam M., Scobie H.M., Sack D.A. (2013). Adaptation of a simple dipstick test for detection of *Vibrio cholerae* O1 and O139 in environmental water. Front. Microbiol..

[34] Proctor L.M. (2016). The National Institutes of Health Human Microbiome Project. Semin. Fetal Neonatal Med..

[35] Proctor L. (2019). Priorities for the next 10 years of human microbiome research. Nature.

[36] The Integrative HMP (iHMP) Research Network Consortium (2019). The Integrative Human Microbiome Project: Dynamic analysis of microbiome-host omics profiles during periods of human health and disease. Nature.

